# MTH1 expression is required for effective transformation by oncogenic HRAS

**DOI:** 10.18632/oncotarget.3447

**Published:** 2015-03-19

**Authors:** Maria G. Giribaldi, Anisleidys Munoz, Katherine Halvorsen, Asmita Patel, Priyamvada Rai

**Affiliations:** ^1^ Department of Medicine, University of Miami Miller School of Medicine, Miami, FL; ^2^ University of California, San Diego, CA; ^3^ OPKA-CURNA, Miami, FL; ^4^ Beckman-Coulter Lifesciences, Indianapolis, IN; ^5^ Sylvester Comprehensive Cancer Center, University of Miami, FL

**Keywords:** MTH1, oncogenic RAS transformation, EMT, glycolysis

## Abstract

Due to sustaining elevated reactive oxygen species (ROS), oncogenic RAS-transformed cells upregulate redox-protective genes, among them the mammalian 8-oxodGTPase, MutT Homolog 1 (MTH1). We previously showed MTH1 abrogates RAS oncogene-induced senescence (OIS) in normal cells and that its inhibition compromises the tumorigenicity of established oncogenic RAS-harboring cancer cells. Here, we investigated how pre-transformation MTH1 levels in immortalized cells influence HRASV12-induced oncogenic transformation. We find MTH1 suppression prior to HRASV12 transduction into BEAS2B immortalized epithelial cells compromised maintenance of high RASV12- and oncogenic ROS-expressing cell populations. Furthermore, pre-transformation MTH1 levels modulated the efficiency of HRASV12-mediated soft agar colony formation. Downstream transformation-associated traits such as the epithelial-mesenchymal transition (EMT) were also compromised by MTH1 inhibition. These collective effects were observed to a greater degree in cells harboring high vs. low RASV12 levels, suggesting MTH1 is required for tumor cells to accumulate RAS oncoprotein. This is significant as, *a priori*, one cannot ascertain whether tumor-promoting adaptations wrought by introducing oncogenic RAS into an immortalized cell are capable of overcoming pre-transformation deficiencies. Our results suggest nucleotide pool sanitization comprises an important transformation-promoting requirement that, if compromised, cannot be adequately compensated post-transformation and thus is likely to affect optimal development and progression of RAS-driven tumors.

## INTRODUCTION

Approximately 30% of all human malignancies sustain activating RAS mutations, which engender aggressive, treatment-resistant tumors. As targeting oncogenic RAS directly has not proven fruitful [1], there is great need to identify additional molecular pathways that promote RAS-driven tumorigenesis which may be targeted in lieu of RAS. Recent thinking has focused on a class of proteins referred to as non-oncogene addictions [2–4]. These proteins, in of themselves, are not involved in oncogenic transformation but enable oncogene-transformed cells to evade tumor-suppressive stresses that lead to cell death or cellular senescence. Among these, human MutT Homolog 1 (MTH1), the major mammalian 8-oxodGTPase has generated much interest as its genetic [5] and pharmacologic [6, 7] targeting was recently shown to inhibit xenograft tumors by a number of different tumor cell lines. MTH1 is an 18 kD nudix pyrophosphorylase enzyme that sanitizes 8-oxodGTP from the nucleotide pool by hydrolyzing it into the monophosphate form which cannot be used by DNA polymerases for genomic incorporation [8].

We have previously shown that MTH1 is elevated in HRASV12-transformed breast cancer cells relative to their non-transformed counterparts and that MTH1 overexpression enables normal cells to overcome oncogene-induced senescence (OIS), the first barrier to oncogenic transformation [9]. Although MTH1 inhibition has tumor-suppressive effects in established RAS-driven tumor cells [5, 9], the role of pre-existing MTH1 levels in modulating the transformation process in immortalized cells, the second step of oncogenic transformation, has not been previously reported. An understanding of this issue is important as it would inform the use of MTH1 as a therapeutic target in RAS-driven tumors.

To address this issue, we utilized BEAS2B immortalized lung epithelial cells that were stably transduced with either a retroviral MTH1-expressing construct or a lentiviral MTH1-targeting shRNA construct. We subsequently co-transduced each of these lines with either a low or high HRASV12-expressing construct to determine how pre-transformation MTH1 levels affect oncogenic transformation and associated phenotypes when RAS oncoprotein levels are low and when the levels accumulate in aggressive tumors, respectively. Our studies show that elevated MTH1 levels enhance RASV12-induced soft agar colony formation and that MTH1 inhibition reduces transformation efficiency and inhibits maintenance of oncogene-induced epithelial-mesenchymal transition (EMT) as well as glycolytic adaptation. These phenomena are more striking in the high RASV12-expressing cells relative to the low RASV12-expressing cells. Thus, our results suggest that elevated MTH1 levels may reflect a predisposition for progression towards more malignant forms of oncogenic RAS-sustaining tumors, which tend to accumulate the RAS oncoprotein.

## RESULTS

### Maintenance of high RASV12-expressing cell populations depends on MTH1 levels

**Figure 1:**
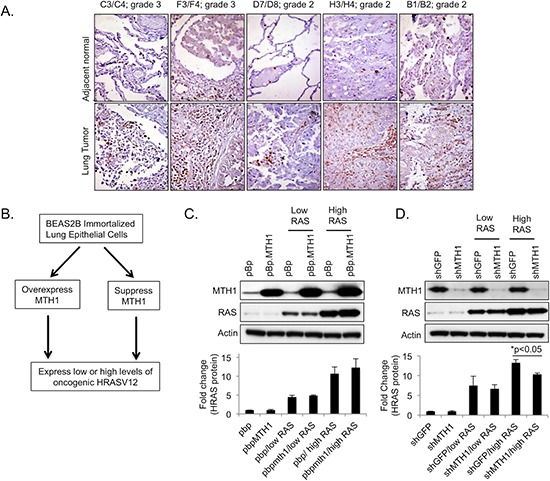
Generation of MTH1/HRASV12 BEAS2B cell lines used in this study **(A)**. MTH1 levels are elevated in nonsmall cell lung tumors relative to adjacent normal tissue. Samples are part of a tissue array obtained from US Biomax. Tissue sections were photographed at 200X. MTH1 expression is seen as brown staining. **(B)**. Schematic showing how cell lines for this study were generated. **(C)**. Western blotting confirming high or low levels of HRASV12 expression in MTH1-overexpressing (pBp.MTH1) and control (pBp) BEAS2B cells. Actin is shown as a loading control. Samples were harvested and lysed approximately 19 days following transduction. Quantitation of HRAS fold-changes (pBp values set to 1) among the samples is shown below the immunoblot images. Fold changes were calculated using loading-normalized signal intensities determined from three independently run blots. **(D)**. Western blotting confirming high and low levels of HRASV12 expression in MTH1-suppressed (shMTH1) and control (shGFP) BEAS2B cells. Actin is shown as a loading control. Samples were harvested and lysed approximately 19 days following transduction. Quantitation of HRAS fold-changes (shGFP values set to 1) among the samples is shown below the immunoblot images and was calculated using loading-normalized signal intensities determined from three independently run blots.

We established that lung tumors display enhanced MTH1 expression relative to adjacent normal lung tissue via immunohistological staining (Figure 1A). We also recently showed that MTH1 levels facilitate proliferation, transformation and tumorigenesis in established RAS-transformed tumor cells [5]. However, we wanted to determine whether pre-transformation MTH1 levels influence oncogenic RAS transformation or whether the pro-malignant effects of MTH1 become important only after transformation. Accordingly, we either stably overexpressed MTH1 via a retroviral expression vector [9] or suppressed MTH1 with a validated lentiviral shRNA construct [9] in BEAS2B immortalized lung epithelial cells. Counterpart cell lines transduced with the appropriate control constructs (pBp retroviral empty vector, shGFP shRNA-expressing vector respectively) were also generated. These constructs were all puromycin-selectable. Subsequently, these cell lines were co-transduced with low HRASV12-expressing (pBh.RAS, hygromycin-selectable) or high HRASV12-expressing (pWZL.RAS, blasticidin-selectable) constructs (Figure 1B). Equivalent amounts of total protein lysates were immunoblotted to validate expression or suppression of these proteins in the various transduced BEAS2B cell lines (Figure 1C, 1D). Note that the exposure times for the MTH1 signal had to be, of necessity, much lower for the MTH1-overexpressing samples to avoid oversaturation of the film due to the strength of overexpressed MTH1 signal.

**Figure 2:**
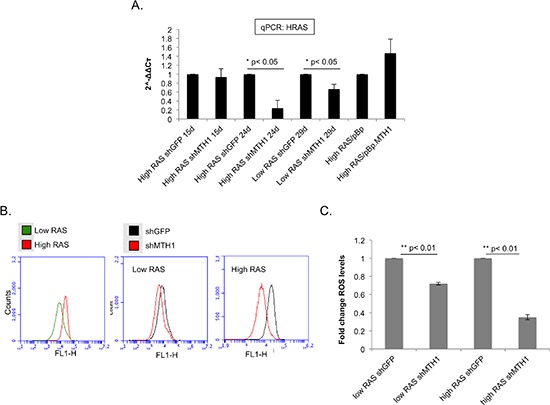
MTH1 expression correlates directly with HRAS expression levels and oncogenic ROS levels **(A)**. MTH1 suppression correlates with HRAS levels in high RASV12-expressing cells. The indicated cell lines were analyzed for HRAS levels via qPCR at the indicated time points following initial transduction. ActinB was used for normalization. Data are derived from two independent experiments, each analyzed in triplicate. **(B)**. Measurement of ROS levels. At approximately three weeks following HRASV12 transduction, the indicated samples were assayed for total cellular ROS levels via CM-DCF-DA staining and flow cytometric analysis on a BD Accuri cytometer. Representative profiles are shown. **(C)**. Quantitation of ROS level changes between indicated shGFP and shMTH1 samples. Values from at least two independent experiments such as those shown in (B) were used for quantitating shMTH1-associated fold-change values relative to shGFP values in each set (shGFP values set to 1).

We noted that at approximately 19 days post-transduction, despite being transduced with the same ectopic RASV12-expressing constructs, the RAS protein levels in the high RASV12 lanes appeared to exhibit a trend of elevation in the MTH1 overexpression background and a significant decrease in the MTH1-suppressed context (Figure 1C, 1D). Therefore, we conducted a quantitative PCR assay for HRAS levels in the different MTH1 and RAS backgrounds, at different time points following RAS transduction (Figure 2A). We found that, in the context of MTH1 suppression, the high HRAS-expressing cells showed a pronounced decrease in RAS levels over time (Figure 2A). The decrease in HRAS levels was much smaller over a comparable time period in MTH1-suppressed low RAS oncoprotein-expressing cells (Figure 2A). Conversely, similar to the immunoblot results in Figure 1C, there was a small perceptible trend of increase in RAS expression in the high RAS MTH1-overexpressing cells (Figure 2A). As MTH1 is unlikely to be altering RAS expression levels directly, this observation suggests that MTH1 expression is important for maintaining high RAS-expressing cell populations.

Because oncogenic HRAS produces ROS, which mediate several aspects of RAS transformation-associated malignant signaling [10–12], we measured whether oncogenic ROS levels also responded to the above-described changes in RAS levels. As expected, the baseline ROS levels were higher for the high RAS oncoprotein-expressing cells relative to the low RAS-expressing cells (Figure 2B). There was no change in ROS levels between the control and MTH1-overexpressing high RAS cells (data not shown). However, there was a significant decrease in cellular ROS levels in the shMTH1 cells relative to the control shGFP counterparts for both the low and high RAS expressing cells (Figure 2B), with an approximately 25% decrease in the low RAS expressing cells and a 65% decrease in the high RAS-expressing cells (Figure 2C). This observation suggests that, following transformation, MTH1 is required to maintain cell populations that exhibit high RAS oncoprotein expression and oncogene-associated ROS levels. The higher degree of ROS reduction in the high RASV12 cells further suggests that MTH1 is required to protect such cells to a greater degree than those that express lower levels of RASV12. As MTH1 has no direct ROS scavenging function, it is likely that the reduced ROS in high RAS/sh MTH1 cells (Figure 2B, 2C) is a result of the reduced RASV12 expression in these cells (Figure 1D, Figure 2A).

### MTH1 expression levels modulate oncogenic RAS-associated anchorage-independent growth

**Figure 3:**
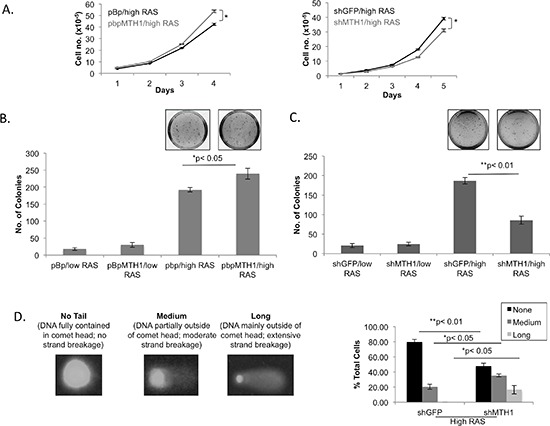
MTH1 levels modulate HRASV12-dependent transformation efficiency **(A)**. Proliferation curves for the indicated samples. Each point was established in triplicate. *p < 0.05 **(B)**. Soft agar colony formation assay from MTH1-overexpressing and control cell lines. Note that in the high RASV12 context, MTH1 promotes colony formation. Representative plate images are shown for high RAS cells. No visual differences in colony number were apparent in low RAS cells. **(C)**. Soft agar colony formation assay from MTH1-suppressed and control cell lines. Note that in the high RASV12 context, MTH1 suppression reduces colony formation. Representative plate images are shown for high RAS cells. No visual differences in colony number were apparent in low RAS cells. **(D)**. DNA strand breaks in the indicated samples were determined via the comet assay. Breaks were categorized via double-blind scoring of tail length as none, medium or long. Examples of each tail type is shown.

We next determined how MTH1 levels affected proliferation, cell death and transformation ability in low or high RAS-expressing cells. We found no MTH1-related differences in either proliferation rates or cell death in the low RAS-expressing cells (data not shown). In the high RAS-expressing cells, there was a small proliferative advantage over 4 days in the MTH1-overexpressing cells and a proliferative defect over 4–5 days in MTH1-suppressed cells (Figure 3A). Minimal cell death was observed over all conditions (data not shown). The relatively small proliferative changes upon MTH1 inhibition likely reflect loss, over time, of the of highest HRASV12-expressing cells, leading to the observed cell population-based decline in HRAS levels.

Because oncogenic RAS levels are correlated with transformative ability [13–15], we also characterized the various cell lines in terms of their soft agar colony formation ability. Soft agar colonies represent the anoikis resistance ability of transformed cells and mimic their ability to form soft tissue tumors. In the case of colony formation, MTH1 overexpression provided a significant advantage in colony formation ability, producing larger numbers of colonies in the high RASV12 expressing cells (Figure 3B). Conversely, MTH1-suppressed BEAS2B cells formed significantly fewer colonies upon expressing high RASV12 than their control shGFP counterparts (Figure 3C). The baseline colony formation was, as expected, lower for the low RASV12 expressing cells and the colony formation ability of the low RASV12 cells were also less affected by pre-existing MTH1 levels (Figure 3B, 3C; pBhRAS samples). Our results suggest that MTH1 levels may be more important for anoikis-resistant growth, a critical feature of RAS transformation, than for adherent proliferation.

Because we had previously found that MTH1 suppression affects cell proliferation via a DNA damage response [16], we assayed the various cell lines for MTH1 suppression-related DNA damage via the alkaline single cell gel electrophoresis or ‘comet’ assay. We found that MTH1 suppression in high RAS cells leads to an increase in the percentage of cells with moderate to high levels of DNA strand breaks (as exemplified by % cells with medium to long comet tails, Figure 3D). Minimal DNA strand breakage was observed in all low RAS cell contexts (data not shown). Based on these results, it is likely that the 13–16% of cells that sustain high degrees of DNA strand breaks (long tails) drop out of the cell population, leading to the observed decline in cell proliferative rates (approximately 20% less in high RAS shMTH1 cells relative to shGFP counterparts; Figure 3A). As the ‘medium tails’ are often indicative of repairable DNA damage, repair intermediates or artefactual damage and based on our prior studies [5, 16], it is unlikely that these contribute to the proliferative defect. However they may be contributing to the overall cellular stress associated with oncogenic RAS and the drop in soft agar colony formation (Figure 3C) through as-yet undefined mechanisms. In the context of MTH1 overexpression, we saw minimal or nonsignificant differences in DNA tails (data not shown). Thus, in the MTH1-overexpressing HRAS-transformed cells, protection from DNA strand breaks alone cannot explain the observed enhancement in soft agar colony formation (Figure 3B).

### MTH1 suppression selects against cells that have undergone oncogenic-RAS induced EMT and glycolytic adaptation

**Figure 4:**
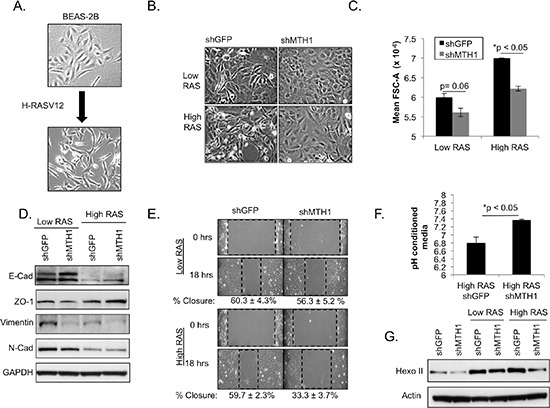
MTH1 suppression compromises maintenance of the RASV12-induced EMT phenotype and reduces expression of the major glycolytic protein, hexokinase II **(A)**. Representative light microscopy images showing induction of an EMT in BEAS2B epithelial cells **(B)**. Representative light microscopy images showing reduced maintenance of EMT phenotype in shMTH1-transduced low- and high-RASV12-expressing cells. **(C)**. Quantitation of cytometric forward scatter area (FSC-A) was carried out as a surrogate for cell size in the indicated samples. FSC-A values from two independently generated profiles were used for quantitation. **(D)**. Molecular markers indicate EMT is generally reduced in HRASV12 shMTH1 cells relative to shGFP counterparts. Western blotting is shown for E-cadherin, N-cadherin, vimentin and ZO-1. **(E)**. MTH1 suppression reduces cell motility in an *in vitro* wound-healing assay. Cell migration edges are marked using dashed rectangles. The width of the rectangle represents the extent of cell migration into the scratched area of the culture, with a narrower width representing greater migration. The percentage of wound closure with standard deviation is noted below representative images. **(F)**. Measurements of pH in conditioned-media from two independent cultures per sample. Samples were seeded at equivalent density and conditioned media was collected approximately 24 hours later. **(G)**. Western blotting to determine hexokinase II (hexo II) levels in the indicated samples.

Introduction of HRASV12 induces an EMT in the epithelial BEAS2B cells (Figure 4A) and this phenotype is believed to contribute to RAS-induced invasion and metastasis [17–19]. We noticed that whereas the HRASV12-transduced BEAS2B shGFP cells exhibited a clear mesenchymal phenotype, a large fraction of the shMTH1 cells retained the cobblestone epithelial morphology of the parental cells. This cobblestone morphology was most pronounced after approximately three weeks of culture in the high HRAS cells (Figure 4B), coinciding with the timepoint at which HRAS expression levels were found to maximally decrease in these cells (Figure 2A). Because the epithelial cells are smaller in size relative to their mesenchymal counterparts, we verified this increase in the epithelial population using the forward scatter (FSC-A) parameter from flow cytometric analysis, which correlates positively with the size and area of cells [20]. We found that, in the high HRASV12-expressing cohorts, there is a greater shift towards lower FSC-A for the shMTH1 cells relative to shGFP cells (Figure 4C), consistent with an increase in epithelial morphology.

The shMTH1-induced reduction in the EMT phenotype was associated with elevated levels of E-cadherin and ZO-1, both epithelial markers [18, 19], relative to the shGFP counterpart cells (Figure 4D). Levels of vimentin, a mesenchymal marker, were decreased with shMTH1 (Figure 4D). The lower baseline vimentin in the high RAS cells vs. the low RAS cells is likely due to RASV12-related suppression [21] even though visually the high RAS cells possess a greater mesenchymal morphology (Figure 4B). N-cadherin, another known mesenchymal marker, was not altered by MTH1 suppression (Figure 4D). Our observations point to MTH1 suppression partially inhibiting the molecular EMT phenotype in the RAS-transformed cell population. Because the EMT phenotype is believed to mark highly motile cells, we also carried out a scratch wound-healing assay and found that the high RASV12-expressing cells exhibited a greater wound closure defect under shMTH1 than the low RASV12-expressing cells (Figure 4E). We note that the extent of MTH1 suppression-associated molecular changes in EMT markers is less fully correlative with the levels of RAS oncoprotein than either the morphological or motility changes. This may reflect a greater functional requirement for the full complement of EMT-associated molecular factors in high RASV12-expressing cells relative to low RASV12-expressing cells, possibly due greater oncogenic dependence in the high RAS cells [22, 23].

RAS-transformed cells are known to undergo the Warburg effect, the ability to utilize glycolysis as their main metabolic pathway for ATP production even under aerobic conditions [24, 25]. Because lactic acid is a major glycolytic substrate, RAS-transformed cells exhibit acidification of their culture media. When we measured the pH of conditioned media from equivalently-seeded, matched shGFP and shMTH1 high RAS samples, we found that the samples from the shMTH1 cells were significantly less acidified than their shGFP counterparts (Figure 4F). We did not see this pronounced change in acidification of media in either the untransformed shMTH1 cells or in the low RAS-transformed shMTH1 cells (data not shown).

**Figure 5:**
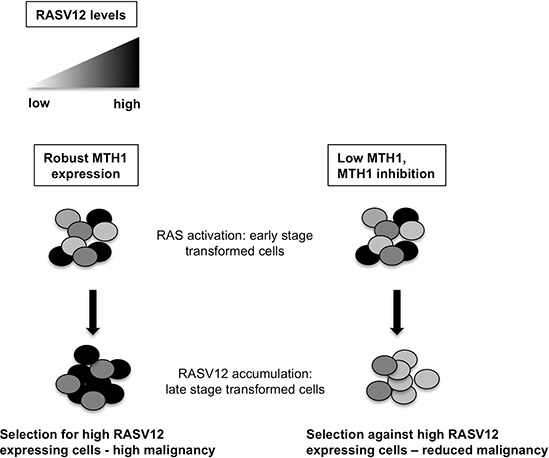
Schematic of MTH1 effects on RASV12-expressing cells Low or suppressed MTH1 inhibits accumulation of high RAS oncoprotein-harboring cells that tend to comprise a malignant, advanced tumor.

In order to determine whether this effect was due to reduced glycolysis which releases lactic acid as a byproduct, we immunoblotted control and RAS-transformed shMTH1 and shGFP samples against hexokinase II [26], a key mediator of aerobic glycolysis that is elevated by oncogenic RAS (Figure 4G). We found that while shMTH1 reduced hexokinase levels slightly in the low RAS-transformed samples, the MTH1 suppression-associated decrease in hexokinase II was much more pronounced in the high RAS samples (Figure 4G). Collectively our results indicate that the MTH1 suppression-induced selection against high oncogenic RAS-expressing cells manifests as an inhibition of downstream pro-malignant traits such as the EMT or glycolytic adaptation (Figure 5).

## DISCUSSION

Here we show that stable overexpression or suppression of MTH1 prior to transformation by HRASV12 affects maintenance of oncogene expression levels, extent of anoikis resistance (as indicated by soft agar colony formation), and components of the EMT and glycolytic adaptation within the RAS-transformed cell population. Our results indicate pre-existing MTH1 levels strongly influence RAS-induced transformation and downstream tumor-promoting phenotypes. This is a significant finding as, *a priori*, one cannot ascertain whether tumor-promoting adaptations wrought by introducing oncogenic RAS into an immortalized cell may be capable of overcoming any pre-transformation deficiencies. Our results suggest that nucleotide pool sanitization comprises an important transformation-promoting requirement that, if compromised, cannot be adequately compensated post-transformation and thus is likely to affect optimal development and progression of RAS-transformed tumors.

A key finding from our study is that low MTH1 levels prevent expansion of cells with high RAS oncoprotein levels and associated ROS levels which mediate oncogenic signaling [27], the EMT phenotype [28] which generates highly metastatic tumors and influences maintenance of cancer stem cell populations [29, 30], and glycolytic adaptation which enables survival under hypoxic tumor growth conditions [31]. This phenomenon puts MTH1 in a relatively unique class of molecules whose inhibition can concurrently compromise multiple oncogenic RAS-associated pathways.

The inhibition of these pathways does not wholly depend on MTH1 suppression-induced DNA strand breaks (Figure 3D), which is lower in extent than what we have previously observed in either MTH1-suppressed fibroblasts [16] or established RAS-driven p53-competent cancer cells [5]. In a prior study, we found that MTH1 suppression similarly affected KRASV12 expression, oncogenic ROS and tumorigenicity in established p53 null or-nonfunctional KRAS-driven lung cancer cells despite an absence of increased DNA strand breaks [5]. Thus our findings suggest that there are complementary or alternate mechanisms, potentially involving RASV12-induced ROS, that can transduce the tumor-suppressive effects of MTH1 inhibition in cancer cells refractory to DNA damage.

RAS-driven malignancy and treatment resistance has been correlated to elevated oncogene levels [32]. Recent studies from our laboratory [5] and others [6, 7] have provided a strong rationale for inhibiting MTH1 in established tumors, particularly RAS-driven tumors. Our findings here suggest inhibiting MTH1 may reduce the malignant potential of oncogenic RAS-activated cells in early stage tumors and potentially limit progression to advanced tumors, which accumulate high levels of RAS oncoprotein (Figure 5).

## METHODS

### Cell culture

BEAS2B cells were obtained from American Type Culture Collection (ATCC) and maintained in DMEM:F12 medium supplemented with 10% fetal bovine serum, 100 units/ml penicillin-streptomycin and 2mM L-glutamine. All culture reagents were obtained for Life Technologies. Cells were maintained at 37°C in a humidified 21% oxygen/5% CO2 Trigas/HERACell incubator.

### Stable transduction of DNA constructs

The pBABE.MTH1 retroviral construct was cloned in our laboratory as described previously [9]. The retroviral pBABE.HRASV12 and pWZL.HRASV12 constructs were a kind gift from Dr. Robert Weinberg's laboratory. The shRNA constructs against GFP and MTH1 have been previously described [9, 16]. Lentiviral or retroviral production in HEK 293T cells and infection of target cells were performed as described previously [33]. Transduced cells were selected in 2.5 μg/ml puromycin-containing, 150 μg/ml hygromycin-containing, or 10 μg/ml blasticidin-containing media for a minimum period of 5–7 days (corresponding to the time taken for untransduced cells to die completely in selection media). Protein overexpression or knockdown was verified via Western blotting.

### Western blotting

Western blotting was carried out as previously described [34]. Approximately 15–25 μg of total protein was run on a 4–12% Bis-Tris pre-cast NuPage gel (Life Technologies) on the Novex immunoblotting system and subsequently transferred onto a section of PVDF membrane (Immobilon, EMD Millipore) at 4°C for either 35V, 2 hours or 15V, overnight. Following transfer, blots were stained with Ponceau reagent to determine even loading and transfer, blocked in 5% milk/0.1% TBST, washed in 0.1% TBST and then probed with antibodies against the following proteins: MTH1 (NB100–109, Novus Biologicals), phospho-Akt (4060, Cell Signaling) total Akt (9272, Cell Signaling), HRAS (sc-520, Santa Cruz Biotech), E-cadherin (610181, BD Transduction Laboratories), ZO-1 (61–7300, Invitrogen/Life Technologies), N-cadherin (610920, BD Transduction laboratories), vimentin (NB200–623, Novus Biologicals), hexokinase-II (2106, Cell Signaling), actin (ab82266, Abcam) and GAPDH (ab9485, Abcam). Following incubation with the appropriate secondary horseradish peroxidase-conjugated antibodies (Amersham), blots were developed using the ECL Plus (GE Healthcare), Lumi-Light (Roche) or Pierce ECL2 (Thermo Scientific) developing solution kits. Western blotting images represent data consistent with a minimum of three independently established sets of samples. Quantitation was carried out using the Gel analysis tool of the ImageJ software (NIH).

### Quantitative PCR

The mRNA preparation from the samples was accomplished as described previously [35]. Briefly, the mRNA was extracted using the Trizol method and cleaned up using the RNeasy kit (Qiagen). Using 1.0 μg of RNA, cDNA was synthesized with the High Capacity cDNA Reverse Transcription kit (Applied Biosystems, cat# 4368814). Samples were run on the following program: 25°C for 10 minutes, 37°C for 2 hours, and 85°C for 5 minutes. The qPCR reaction was set up with 1.0 μl of cDNA and 20X Taqman probes and Taqman Universal PCR Master Mix (Applied Biosystems cat# 4324018) in a 15 μl total volume. Samples were run in triplicate on an Applied Biosystem Real-Time machine using a StepOne program consisting of 95°C for 10 minutes and 40 cycles of the following: 95°C for 15 seconds and 60°C for 1 minute. Gene expression levels were calculated using the 2^−ΔΔCT^ method [36]. The following Taqman probes were used: Hs00978050_g1 (HRAS) and Hs99999903_m1 (Actin-B).

### Soft agar colony formation assay

Noble agar (BD Biosciences) at a 2.4% stock concentration was added to a solution containing DMEM, 20% FBS and 200 units of streptomycin:penicillin/fungizone to yield a 0.6% bottom agar solution. A 0.3% top agar solution containing the suspended target cells was layered over pre-solidified bottom agar in 6 cm dishes. The assay was set up in triplicate for each sample, with 10^4^ cells seeded per dish. After the upper layer solidified, soft agar dishes were transferred to a humidified 37°C tissue culture incubator and examined the following day to ensure only single cells were seeded through the agar. Every six days, approximately 100–200 μl of fresh DMEM media per dish was added dropwise to keep the agar hydrated. Colonies were scored three weeks post-seeding using a visual counting grid under a light microscope over 6–8 random fields per dish per sample.

### Cell proliferation assay

Either 1 × 10^5^ or 2 × 10^5^ cells were seeded on Day 0. Cells were counted in triplicate on indicated days via a hemocytometer and media was refreshed on counterpart dishes not used for counting.

### Comet assay

The comet assay was carried under alkaline conditions out as per kit instructions (Trevigen) and as described previously [5]. Data represent averages from two independent experiments, each carried out in duplicate. A minimum of 50 cells was counted per sample.

### Wound-healing assay

Confluent 10 cm dishes of cells were scored across the pre-marked dish diameter with a 1000 μl pipette tip. Media was changed gently to remove floating cells and representative images were acquired right after the scratch wound and then after 18 hours. To determine area of wound closure, the scratched area edges were fitted to a rectangle whose dimensions were used to calculate open areas and to determine the percentage of closure by normalizing wound area after 18 hours to the initial wound area.

### Flow cytometry and detection of ROS levels

The assay was carried out as described previously [16]. Briefly, the indicated cells at equivalent numbers were collected through trypsination, washed in ice-cold 1X Hank's buffered saline solution (HBSS) and incubated with 10 μM freshly prepared 5- (and-6)-chloromethyl-2′, 7′-dichlorofluorescein diacetate (CM-DCF-DA; Molecular Probes/Life Technologies, C6827) for 25 min at 37°C. The cells were then washed and resuspended in 1X HBSS/2% FBS prior to analysis via fluorescence-activated cell sorting (FACS). Flow cytometric analysis was conducted on an Accuri C6 cytometer (BD Biosciences). The x-axis represents FITC channel (FL1) fluorescence intensity in log-scale and the y-axis represents the number of cell counts. Quantitation of fluorescent signal was carried out via automated mean FL1 fluorescence intensities provided by the BD Accuri Cytometer software.

### Immunohistochemistry of tumor sections

The formalin-fixed tissue array (LC1006, US Biomax) comprising matched NSCLC tumor and matched adjacent normal tissue was deparaffinized in xylene and hydrated with alcohol prior to staining. For the xenograft tissues, a section of fresh tumor tissue was placed in a tissue cassette and fixed in formalin overnight prior to processing for immunohistological analysis. For immunostaining, endogenous peroxides were blocked using 3% H_2_O_2_ in methanol for 5 min. Antigen retrieval was done by incubating sections in hot 10 mmol/L citrate buffer (pH, 6.0) for 20 min. Sections were blocked in normal goat serum and stained for human MTH1 (Novus Biologicals) using 1/150 dilution followed by a biotinylated anti-rabbit IgG secondary antibody (Vector Laboratories, Burlingame, CA). Signal was developed via an ABC reagent and DAB peroxidase (Vector Laboratories). Images were photographed using a Nikon Microphot-FXA microscope and a Nikon Coolpix 4300 digital camera.

### Statistical analysis

Data are represented as mean ±standard deviation. Significance was determined via a two-tailed Student's *t*-test, with *p* < 0.05 considered significant.
